# Web-Based Social Networks of Individuals With Adverse Childhood Experiences: Quantitative Study

**DOI:** 10.2196/45171

**Published:** 2023-05-30

**Authors:** Yiding Cao, Suraj Rajendran, Prathic Sundararajan, Royal Law, Sarah Bacon, Steven A Sumner, Naoki Masuda

**Affiliations:** 1 Department of Systems Science and Industrial Engineering Binghamton University State University of New York Binghamton, NY United States; 2 Tri-Institutional Program in Computational Biology and Medicine New York, NY United States; 3 Department of Biomedical Engineering Georgia Institute of Technology Atlanta, GA United States; 4 Division of Injury Prevention National Center for Injury Prevention and Control Centers for Disease Control and Prevention Atlanta, GA United States; 5 Office of Strategy and Innovation National Center for Injury Prevention and Control Centers for Disease Control and Prevention Atlanta, GA United States; 6 Department of Mathematics State University of New York at Buffalo Buffalo, NY United States

**Keywords:** adverse childhood experience, ACE, social networks, Twitter, Reddit, childhood, abuse, neglect, violence, substance use, coping strategy, coping, interpersonal connection, web-based connection, behavior, social connection, resilience

## Abstract

**Background:**

Adverse childhood experiences (ACEs), which include abuse and neglect and various household challenges such as exposure to intimate partner violence and substance use in the home, can have negative impacts on the lifelong health of affected individuals. Among various strategies for mitigating the adverse effects of ACEs is to enhance connectedness and social support for those who have experienced them. However, how the social networks of those who experienced ACEs differ from the social networks of those who did not is poorly understood.

**Objective:**

In this study, we used Reddit and Twitter data to investigate and compare social networks between individuals with and without ACE exposure.

**Methods:**

We first used a neural network classifier to identify the presence or absence of public ACE disclosures in social media posts. We then analyzed egocentric social networks comparing individuals with self-reported ACEs with those with no reported history.

**Results:**

We found that, although individuals reporting ACEs had fewer total followers in web-based social networks, they had higher reciprocity in following behavior (ie, mutual following with other users), a higher tendency to follow and be followed by other individuals with ACEs, and a higher tendency to follow back individuals with ACEs rather than individuals without ACEs.

**Conclusions:**

These results imply that individuals with ACEs may try to actively connect with others who have similar previous traumatic experiences as a positive connection and coping strategy. Supportive interpersonal connections on the web for individuals with ACEs appear to be a prevalent behavior and may be a way to enhance social connectedness and resilience in those who have experienced ACEs.

## Introduction

### Background

Adverse childhood experiences (ACEs) are preventable, potentially traumatic events that occur in childhood (ages of 0-17 years), such as being neglected, experiencing or witnessing violence, and having a family member attempt or die by suicide. Also included are aspects of a child’s environment that can undermine their sense of safety, stability, and bonding, such as growing up in a household with substance use; mental health problems; or instability because of parental separation or incarceration of a parent, sibling, or other member of the household [1,2]. These examples do not comprise an exhaustive list of childhood adversity as there are other traumatic experiences that could affect health and well-being. ACEs often occur together; can result in toxic stress; and are associated with a wide range of adverse behavioral, health, and social outcomes, including substance use, depression, obesity, lower education and earning potential, and chronic diseases such as heart disease and cancer. ACEs are preventable. The prevalence of ACEs is estimated to be as high as 60% in the United States [3-5] and worldwide [4]. With the advent of the COVID-19 pandemic in early 2020 and the following economic stress and instability in the United States, there has been an increase in stress in parenting [6], which may contribute to increased risk of ACEs [7,8].

Connecting youth with caring adults and activities is an evidence-based strategy for the primary prevention of ACEs. An extension of this strategy is that connectedness and social support have been suggested to play an important role in mitigating negative impacts of ACEs across the life span. In fact, longitudinal studies have shown that individuals with ACEs tend to have less interpersonal social support than those without ACEs when they become adults [9-11]. Although social support as a mitigating factor in various health problems has been studied for decades, the definition of social support can vary [12,13]. It has been suggested that social networks of individuals (ie, how they are connected with each other through social ties and embedded in social groups) can be a powerful form of social support [14,15]. Understanding the characteristics of social networks of individuals who discuss their ACEs and their health is useful for identifying where and how social support can be improved.

Studies of social networks of individuals reporting ACE exposure have largely focused on examining the number and type of other individuals in close physical proximity with whom they directly interact and the quality of such social ties [16,17]. However, social networks are not only defined by numbers and proximity but also by the relationships between individuals. Particularly in the modern, internet-enabled culture, individuals are embedded in a larger social network through which information and support can flow in more expansive ways than previously possible [18,19]. Social media is universally used to discuss various topics, including health, and ACEs are no exception [20]. Social media information has also been used to estimate the mental health status of individuals [21-24]. These and other results suggest opportunities for researchers to use social media information to better understand conversations about ACEs, understand health-related information pertaining to ACEs, and enhance social support among those with ACEs. Previous research in other health topics, including major depression [25-27], suicide ideation [25,28], anxiety disorder [29], and schizophrenia [30], have revealed that the characteristics of an individual’s web-based social network bear an association with aspects of health status.

### Objectives

Motivated by this, we used data from 2 social media platforms to examine the web-based social networks of individuals reporting ACEs and discuss implications for public health.

## Methods

### Overview

This work involved a multistep pipeline to first create a classifier to identify ACE content from social media posts, deploy the classifier to a large body of social media posts, and compute network statistics from such information. In Textbox 1, we provide a short description of the terms introduced in the following subsections.

**Glossary of terms.**

**Accuracy**

- This is a measure of classification performance, defined by the fraction of the correct prediction.

**Precision**

- This is a measure of classification performance, defined by the fraction of true positives among all samples classified as positive.

**Recall**

- This is a measure of classification performance, defined by the fraction of true positives among all positive samples.

***F*_1_-score**

- This is the harmonic mean of the precision and recall.

**Area under the curve**

- This is a measure of classification performance calculated as the area under the receiver operating characteristic curve, which is computed based on true positives, false positives, and their numeric scores.

**Adverse childhood experience (ACE) mention score**

- This is the output of our classifier for a tweet input. It ranges from 0 to 1, and a larger score suggests that the tweet is more strongly related to ACEs.

**ACE alignment index**

- A score for a Twitter user quantifying how strongly the user is likely to be associated with ACEs. We calculate the ACE alignment index for user based on all the tweets of *u*. We call *u* an ACE individual if *u*’s ACE alignment index is at least 0.5.

**Sentiment analysis**

- This is a natural language processing technique to classify text into positive, neutral, and negative groups.

**Edge**

- This is the following relationship between 2 Twitter users represented by an arrow. If user *u* follows user *u*’, then there is an arrow (ie, directed edge) pointing from *u* to *u*’.

**Egocentric network**

- This is the local network around user *u*.

**Root user**

- These are the sampled Twitter users for which we constructed and analyzed egocentric networks.

**Reciprocity**

- This is the fraction of edges owned by user *u* that are bidirectional (ie, *u* follows *u*’, and *u*’ follows *u*).

**Local clustering coefficient**

- This is the measure of the abundance of triangles containing user *u*. The local clustering coefficient for user *u* ranges from 0 to 1.

**Homophily**

- This is the tendency for similar nodes to be adjacent (ie, next to each other) in a network. We specifically examined whether ACE users tended to be adjacent to other ACE users.

### Reddit Data

To first construct a machine learning–based, automated classifier for self-reported ACE disclosures, we used a transfer learning approach commonly used in web-based data research [31]. Using the Pushshift.io Reddit application programming interface (API) [32], we downloaded Reddit data from 2 subreddits: r/raisedbynarcissists and r/internetparents. Subreddit r/raisedbynarcissists is a support group for people raised by abusive parents and, hence, contains many posts detailing experiences of ACEs. Subreddit r/internetparents is structured similarly to r/raisedbynarcissists but focuses on generally positive childhood events and experiences. We used these 2 subreddits as the explicit labels to build a binary supervised learning classifier with r/raisedbynarcissists as the positive class, associated with the presence of ACEs, and r/internetparents as the negative class, associated with the absence of ACEs. Note that we could not use Twitter data for training a classifier as tweets do not have ACE-related labels. We collected all available posts within the 2 subreddits between December 25, 2020, and March 31, 2022, totaling 49,044 posts from r/raisedbynarcissists and 21,712 posts from r/internetparents. We used the titles of the post to train the classifier as our investigation revealed that post titles are similar in length to tweets and contain sufficient information about the ACE experienced.

Before submitting the Reddit post titles to the training of the classifier, we cleaned the post titles from both subreddits using the Python-based regular expression (RegEx; Python Software Foundation) and Natural Language Toolkit (NLTK Team) [33] libraries. Our natural language processing (NLP) pipeline to clean text included dropping duplicate post titles, expanding abbreviations, and removing special characters, among others. We deleted all post titles with ≤5 words to improve the training of the classifier. There were, in total, 70% (22,950/32,694) of posts from r/raisedbynarcissists and 30% (9744/32,694) of posts from r/internetparents that were ultimately used in the model. Details on the text preprocessing are available on GitHub [34].

### Convolutional Neural Network Classifier

#### Training of the Classifier

We used a convolutional neural network (CNN) for our classifier. CNNs have successfully been applied to image and text processing [35,36]. CNNs have also been used in detecting mental health conditions from Reddit data [37,38]. CNNs work by modeling hierarchical complicated patterns using smaller and simpler patterns. Convolutional layers along with the max-pooling layer allow the CNNs to learn useful word representations while enhancing their computational efficiency.

We trained a CNN using the Reddit post title as the input and the class label as the teacher. As there were more data in the positive class than in the negative class, we selected samples from the positive class uniformly at random to make the number of samples in the positive class be the same as that in the negative class. This preprocessing is necessary for the training and testing of the CNN [39]. Then, for each of the 2 subreddits, we used 72% of data selected uniformly at random (ie, post titles) as training data, 8% as validation data, and 20% as the test data. To input post titles of different lengths to the CNN, we set the length of the input in terms of the number of words to the largest one among all the post titles, which was equal to 45 words after data cleaning. When an input post title was shorter than this length, we padded zeros after the post title to make the total length 45 words. Note that almost all the tweet samples (158,610/158,706, 99.94%) that we collected from Twitter, which we used as input to the trained CNN in the following analyses, were shorter in length than the maximum input length allowed for the CNN (ie, 45 words after cleaning). For any tweets of >45 words after cleaning (ie, at most, 45 words after cleaning), we fed the first 45 words to the CNN. We used Keras [40] with TensorFlow (Google Brain Team) [41] as a back end to set up the neural network structure and train it.

The trained CNN is a softmax classifier, which outputs a value between 0 and 1. The output value is the ACE mention score value (see the *ACE Mention Score for Tweets and ACE Alignment Index for Twitter Users* section for the ACE mention score) and represents the probability that the input text contains references to an ACE. If the output is >0.5, the classifier judges the input to be associated with an ACE, which is part of the information used for training the CNN.

The training of the CNN also required a word-embedding matrix. Word embeddings can capture the semantic meaning of words by converting them into numeric vectors [42], and for this, we used Global Vectors for Word Representation (GloVe) [43]. GloVe is a widely used mapping from words to vectors, equivalent to a word-embedding matrix whose rows and columns correspond to the words and the vector’s components, respectively.

#### Measures of Classification Performance

To evaluate the classification performance of the trained CNN on the test data, we calculated the following 5 measures of classification performance [44]. To explain the 5 quantities, we denote the number r/raisedbynarcissists posts that were correctly classified into the ACE-positive group as true positives (TPs), the number r/internetparents posts that were correctly classified into the negative group as true negatives (TNs), the number r/internetparents posts that were incorrectly classified into the positive group as false positives (FPs), and the number r/raisedbynarcissists posts that were incorrectly classified into the negative group as false negatives (FNs).

The accuracy is equal to the fraction of the correct prediction, that is, *(TP + TN) / (TP + TN + FP + FN)*. The precision is given by *TP / (TP + FP)*. The recall is given by *TP / (TP + FN)*. The *F*_1_-score is the harmonic mean of the precision and recall, that is, *2 × (Precision) × (Recall) / (Precision + Recall)*. Finally, we measured the area under the receiver operating characteristic curve (AUC). The receiver operating characteristic curve is the trajectory of the FPs and TPs, with FPs on the horizontal axis and TPs on the vertical axis, when we gradually increase the threshold for classification in terms of the output value of the CNN from 0 to 1.

Note that we fixed the threshold to 0.5 to actually classify the Reddit post titles and calculate the accuracy, precision, recall, and *F*_1_-score. Therefore, the definition of FP and TP for calculating the AUC is different from that for calculating these 4 measures. A large AUC value indicates a good performance of binary classification.

### ACE Mention Score for Tweets and ACE Alignment Index for Twitter Users

#### ACE Mention Score

We used the Twitter Intelligence Tool (TWINT) [45] to collect publicly available tweets, excluding retweets. We queried the tweets via the keywords explained in the *Egocentric Networks of ACE and Non-ACE Twitter Users* section (see also Textbox 2). The time frame used for the collection of the tweets was the same as that used for the Reddit posts (ie, from December 25, 2020, to March 31, 2022). We restricted ourselves to English-language tweets and otherwise did not use other filters. We then used the previously described classifier to score tweets containing >5 words after cleaning (see the *Reddit Data* section for the cleaning procedure). We refer to the computed output as the ACE mention score of the tweet, and it represents the probability that the tweet contains references to an ACE.

**Keyword lists for sampling Twitter users. The vertical bars between the words represent OR. OD abbreviates overdose.**

**Adverse childhood experience (ACE)**

- (my mother | my father | my mom | my dad | my guardian) AND *S*

**Non–ACE-1**

- (my mother | my father | my mom | my dad) AND NOT *S*

**Non–ACE-2**

- (school | basketball | game | dog | cosplay | shopping) AND NOT *S*

***S***

- (abuse | neglect | jail | prison | substance use | substance misuse | substance abuse | overdose | OD | drug addiction | parental separation | divorce)

#### ACE Alignment Index

For each Twitter user *u* that was the author of any of the collected tweets and had at least 30 tweets with >5 words after cleaning, we then submitted each tweet posted to the CNN classifier, obtaining its ACE mention score value. We then defined the top 10% value of the ACE mention score calculated from all tweets of *u* as *u*’s ACE alignment index. The intuition behind this definition is that individuals reporting ACEs would tweet about ACEs at least 10% of the time. The ACE alignment index ranges from 0 to 1 as the ACE mention score of each tweet ranges from 0 to 1.

**Figure 1:**
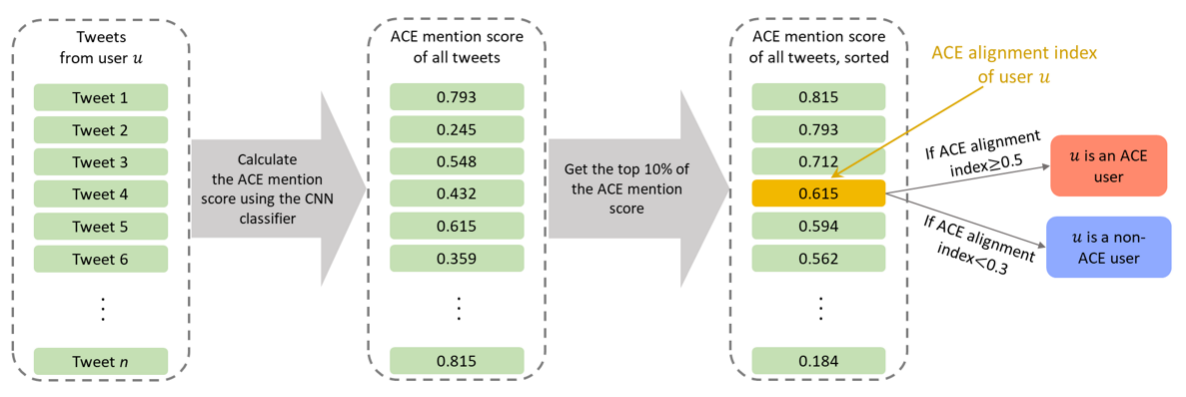
Schematic showing calculation of the adverse childhood experience (ACE) alignment index of a Twitter user *u*. CNN: convolutional neural network.

If *u*’s ACE alignment index is ≥0.5, we say that *u* is an ACE individual. We manually inspected the ACE alignment index of the sampled Twitter users and their tweets to conclude that, although the threshold value of 0.5 for defining the ACE individual was reasonable, labeling all the individuals whose ACE alignment index was <0.5 as non-ACE was inappropriate as there were many equivocal cases. Therefore, we defined non-ACE users as those whose tweets with >5 words after cleaning had an ACE mention score of <0.3. We show in Figure 1 the entire process of calculating *u*’s ACE alignment index and classifying *u* into the ACE or non-ACE category.

### Sentiment Analysis

We calculated a standard sentiment score using a pretrained rule-based analysis model called Valence Aware Dictionary and Sentiment Reasoner (VADER) [46] to determine the sentiment of all tweets with >5 words after cleaning posted by each Twitter user *u*. The sentiment score, denoted by *s*, ranged between –1 and 1, and *s* ≤ –.05, –.05 < *s* < .05, and *s* ≥ .05 indicated negative, neutral, and positive sentiment, respectively.

### Egocentric Networks of ACE and Non-ACE Twitter Users

**Figure 2:**
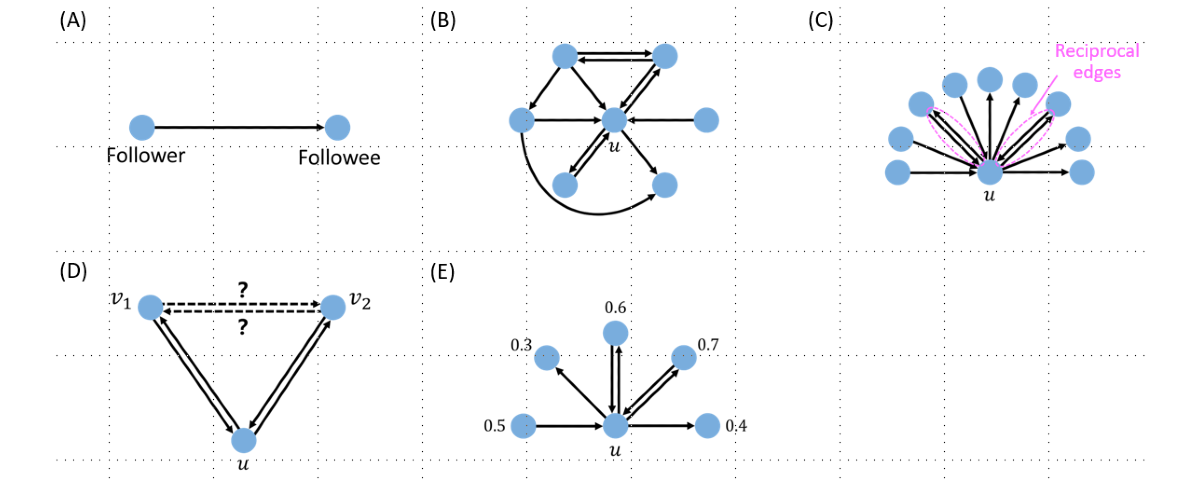
Schematic of the follow network and indexes. (A) A directed edge, which points from a follower to a followee by definition. (B) A hypothetical example of the egocentric network of a root user *u*. (C) Reciprocity indexes. Root user *u* has *m*_1_=5 followers and *m*_2_=6 followees. As they have 2 reciprocal neighbors, one obtains *r*_1_=2/5 and *r*_2_=2/6=1/3. (D) Local clustering coefficients. Users *v*_1_ and *v*_2_ are reciprocal neighbors of *u*. If *v*_1_ and *v*_2_ are reciprocal neighbors of each other, the reciprocal edge contributes to local clustering coefficients *C*_1_ or *C*_2_ as well as to *C*_1_^*'*^ or *C*_2_^*'*^. If *v*_1_ and *v*_2_ are only unidirectionally connected (ie, from *v*_1_ and *v*_2_ or vice versa but not both), the unidirectional edge contributes to *C*_1_^*'*^ or *C*_2_^*'*^ but not to *C*_1_ or *C*_2_. (E) Calculation of the average adverse childhood experience (ACE) alignment index over the followers or followees. The numbers shown are the ACE alignment index. User *u* has 3 followers, and 〈α〉_follower_=(0.5+0.6+0.7)/3. User *u* has 4 followees, and 〈α〉_followee_=(0.3+0.6+0.7+0.4)/4=0.5.

We generated egocentric follow networks for ACE and non-ACE individuals. Nodes of a follow network are Twitter users. Each edge represents a following relationship, is directed from the follower to the followee, and is unweighted, as schematically shown in Figure 2A. We first needed to sample users from each category (ie, ACE and non-ACE) whose egocentric networks we built. We call these users *root users*. To obtain root users, we ran a keyword search on all public tweets. With the aim of sampling tweets related to ACEs, we used the keyword list labeled “ACE” in Textbox 2. Notably, we added *my* before each word related to a parent or guardian. This is because, without *my*, we obtained a large fraction of institutional and individual accounts that tweeted about ACEs but they themselves did not experience ACEs [20]. With *my*, we intended to sample users who had self-reported ACEs. We then filtered the sampled users according to the following criteria. First, we examined public Twitter profiles and tweets to remove institutional and individual accounts that were advocating for or supporting ACEs but had not tweeted about personal experiences. Second, for the ACE category, we obtained root users for whom the ACE alignment index was at least 0.5 from the steps described in the *ACE Mention Score for Tweets and ACE Alignment Index for Twitter Users* section.

To construct diverse samples of non-ACE root users for comparison purposes, we used each of the 2 keyword lists, named non–ACE-1 and non–ACE-2, shown in Textbox 2. The non–ACE-1 keyword list is informed by the ACE keyword list. To construct non–ACE-1, we removed keywords related to events associated with ACEs, such as *jail* from the ACE keyword list. We also removed *my guardian* from the ACE keyword list to generate non–ACE-1 as *my guardian* suggests that sampled individuals have been separated from their biological parents and undergone foster care placement, which itself is an ACE. The non–ACE-2 keyword list is an arbitrarily chosen set of keywords that are not related to parenting. We filtered the users sampled using either the non–ACE-1 or non–ACE-2 keyword list in the same manner as the case of sampling ACE individuals using the ACE keyword list except that we retained the users whose tweets had an ACE mention score of <0.3. We refer to the final sets of root users obtained using these 2 keyword lists as non–ACE-1 and non–ACE-2 individuals.

For each root user *u* that belonged to either the ACE, non–ACE-1, or non–ACE-2 group, we collected their followers and followees (ie, users that a user follows, which Twitter officially calls “following”) using the Python library Tweepy [47]. We also collected the follow edges between *u*’s followers and followees if they existed. Owing to the rate limit of the Twitter API, we only sampled up to 100 followers and 100 followees of each root user and part of connectivity between pairs of them, as we describe in the *Network Indexes* section. These connectivity data define egocentric networks of the root users (see Figure 2B for an example; *u* represents a root user).

### Network Indexes

Apart from the number of followers and followees, we measured the following 3 quantities for the egocentric network of each root user.

#### Reciprocity

In directed networks, reciprocal edges (ie, bidirectional edges) are considered to represent stronger relationships than unidirectional edges [48,49]. In the case of the follow relationship on Twitter, a reciprocal edge represents reciprocal following between 2 individuals (Figure 2C). Such pairs of individuals may be friends with each other. To compare the reciprocity of follow edges between ACE and non-ACE individuals, we measured 2 reciprocity indexes for individuals defined as follows. For a given root user *u*, we sampled *u*’s 100 followers uniformly at random. If *u* had <100 followers, we sampled all of *u*’s followers. In either case, we denote using *m*_1_ the number of the sampled followers. Then, among the *m*_1_ followers of *u*, we counted the number of those whom *u* followed back. We refer to such individuals as reciprocal neighbors of *u*. In other words, *u* both follows and is followed by each of their reciprocal neighbors. In the example shown in Figure 2C, the root user has *m*_1_=5 followers and 2 reciprocal edges. We then define reciprocity *r*_1_ as the number of the reciprocal neighbors of *u* divided by *m*_1_. The root user shown in Figure 2C has *r*_1_ = 2/5. We only sampled up to 100 followers because of the rate limit of the Twitter API. We avoided calculating *r*_1_ when *u* had <5 followers as the calculated *r*_1_ value is considered unreliable when *m*_1_ is small.

Similarly, we sampled 100 followees of *u* selected uniformly at random or all the followees if *u* had <100 followees. We denote using *m*_2_ the number of the sampled followees. Reciprocity *r*_2_ is equal to the number of the reciprocal neighbors of *u* divided by *m*_2_. The root user shown in Figure 2C has *m*_2_=6 followees and, therefore, *r*_2_ = 2/6 = 1/3. We avoided calculating *r*_2_ when *u* had <5 followees. Thus, *r*_1_ is a reciprocity measure among a root user’s followers, and *r*_2_ is a reciprocity measure among a root user’s followees.

Both *r*_1_ and *r*_2_ range from 0 to 1. A unified measure of reciprocity for individual *u* would be the number of reciprocal edges divided by the number of any edges owned by *u* [49]. However, its computation requires collecting all the followers and followees, which is impossible because of the rate limit of the Twitter API. Therefore, we instead measured *r*_1_ and *r*_2_ for each root user.

#### Local Clustering Coefficients

The presence of triangles around an individual *u* suggests that *u* belongs to a group of at least 3 individuals, and such groups may provide social support to *u* [25,28,50,51]. Therefore, we measured the abundance of triangles around each root user using the sample local clustering coefficients defined as follows. Consider a root user *u*. We then obtain the subset of the *m*_1_ followers of *u* that *u* follows back. This subset defines a set of reciprocal neighbors of *u*. We then consider 2 reciprocal neighbors of *u* in this set, denoted using *v*_1_ and *v*_2_, and ask whether *v*_1_ and *v*_2_ are reciprocal neighbors of each other (Figure 2D). If they are (ie, if *v*_1_ follows *v*_2_ and *v*_2_ follows *v*_1_), then *u*, *v*_1_, and *v*_2_ form a triangle in which each pair of individuals is connected by reciprocal edges. We define *u*’s local clustering coefficient, denoted using *C_1_*, as the fraction of (*v*_1_, *v*_2_) pairs that are reciprocal neighbors. We also measure a weaker version of the local clustering coefficient, denoted using *C_1_^’^* which only requires that *v*_1_ and *v*_2_ are adjacent by 1 follower edge in either direction (ie, either *v*_1_ follows *v*_2_ or vice versa). We restricted *v*_1_ and *v*_2_ to be *u*’s reciprocal neighbors, not just followers or followees, as the local clustering coefficient is primarily used for undirected networks. We avoided calculating *C_1_* and *C_1_^’^* when *u* had <5 followers. Note that 0 ≤ *C_1_* ≤ *C_1_^’^* ≤ 1.

We repeated the same measurements using a different set of reciprocal neighbors of *u*, which was the subset of the *m*_2_ followees of *u* that followed back *u*. We denote the thus calculated local clustering coefficients, depending on whether *v*_1_ and *v*_2_ are reciprocally connected or at least unidirectionally connected, using *C_2_* and *C_2_^’^*, respectively. We avoided calculating *C_2_* and *C_2_^’^* when *u* had <5 followees.

#### Homophily

Finally, we hypothesized that ACE individuals tend to be adjacent to other ACE individuals, presenting homophily. To test this hypothesis, we measured the fraction of ACE neighbors for ACE root users and non-ACE root users. To this end, for each root user *u*, we used their *m*_1_ followers sampled for the calculation of the reciprocity and local clustering coefficients. We calculated the ACE alignment index of each follower whose ACE alignment index could be calculated (ie, those with at least 30 tweets with >5 words after cleaning) and took the average of all such followers. This average, denoted using 〈α〉_follower_, ranges from 0 to 1 and defines the average ACE alignment index of *u*’s followers. In the example shown in Figure 2E, the root user *u* has 3 followers, and 〈α〉_follower_ = (0.5 + 0.6 + 0.7) / 3 = 0.6. We avoided calculating 〈α〉_follower_ for the root users *u* when we could not calculate the ACE alignment index for any of *u*’s followers (ie, when none of *u*’s followers had at least 30 eligible tweets). We also measured the average ACE alignment index of *u*’s followees, denoted using 〈α〉_followee_, in the same manner. For example, the root user *u* in Figure 2E has 4 followees, and 〈α〉_followee_ = (0.3 + 0.6 + 0.7 + 0.4) / 4.

We also compared the average ACE alignment index of the reciprocal neighbors of root users *u* and of nonreciprocal followers of *u*. For example, individual *u* shown in Figure 2E has 2 reciprocal followers, and their average ACE alignment index is (0.6 + 0.7) / 2 = 0.65. The same individual has just 1 nonreciprocal follower whose (average) ACE alignment index is 0.5. If the former tends to be larger than the latter, as in this example, then *u* tends to follow back other ACE individuals more than non-ACE individuals.

### Ethical Considerations

Approval by an ethics committee was not needed for this study as no intervention or trial occurred and the data were open to the public from Reddit and Twitter. Obtaining informed consent was unnecessary as there was no direct contact between the sampled Reddit or Twitter users. The study data were deidentified and stored securely. The Reddit data were anonymously collected and were not published in any source. Sharing the raw Twitter data set is prohibited under Twitter’s policy. The information that we share regarding Reddit and Twitter data is only the reported keywords for querying users and the retrieval time frames to allow others to replicate qualitatively the same data collection as ours.

## Results

### Performance of the CNN Classifier

Our CNN trained using the Reddit data had an average accuracy of 82.78%, precision of 86.32%, recall of 78.07%, *F*_1_-score of 81.99%, and AUC of 91.34%. Using the same Reddit data set, we also trained the Bidirectional Encoder Representations from Transformers (BERT), which is a machine learning technique specialized to NLP [52]. However, its performance on the Reddit data was worse than that of the CNN (accuracy: 80.46%; precision: 80.93%; recall: 79.50%; *F*_1_-score: 80.21%; AUC: 89.31%). Therefore, we used the CNN in the following analyses to classify tweets. However, we verified that the results remained similar when we used the BERT trained with the Reddit data to classify tweets (see the *Analysis of Twitter Data Using the BERT Classifier* section in Multimedia Appendix 1 for details).

### Sampling ACE and Non-ACE Twitter Users

**Figure 3:**
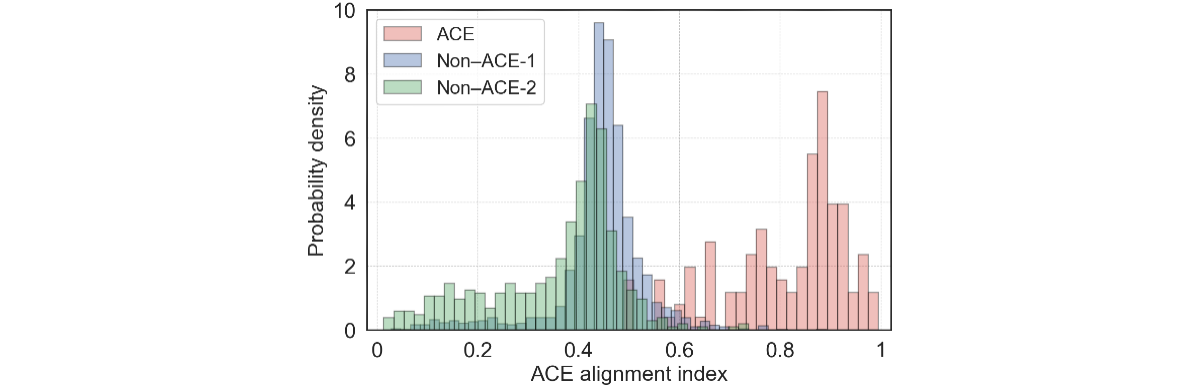
Distribution of the adverse childhood experience (ACE) alignment index for the 3 groups of Twitter users.

We sampled Twitter users using any of the 3 lists of keywords shown in Textbox 2 with the aim of sampling ACE and non-ACE individuals. We show in Figure 3 the distribution of the ACE alignment index for the 3 groups of individuals (ie, those sampled using the ACE keyword list, those sampled using the non–ACE-1 keyword list, and those sampled using the non–ACE-2 keyword list). Note that individuals sampled using the ACE keyword list may be non-ACE, and vice versa. As expected, we found that the ACE alignment index for the individuals sampled using the ACE keyword list tended to be larger than that of those sampled using either of the 2 non-ACE keyword lists (ACE vs non–ACE-1: *P*<.001; ACE vs non–ACE-2: *P*<.001; Mann-Whitney-Wilcoxon test, 2-sided, Bonferroni corrected, and including the comparison between non–ACE-1 and non–ACE-2; we used the same statistical test in the following group comparison analyses). In particular, most users sampled using the ACE keyword list (123/126, 97.6% of the sampled users) had an ACE alignment index of at least 0.5 (mean 0.81, SD 0.12). Those users were ACE individuals by definition.

For validation, we also sampled Twitter users using a previously published list of keywords for ACEs [20]. With this keyword list, the ACE alignment index for the sampled individuals was 0.55 (SD 0.10), and 81.9% (113/138) of individuals had an ACE alignment index of at least 0.5. These numbers are substantially smaller than those for the individuals sampled using our ACE keyword list (*P*<.001). Furthermore, the fraction of institutional accounts, which we needed to manually remove, was much larger using the previously published ACE keyword list (19/138, 13.8%) than using our ACE keyword list (3/126, 2.4%). Therefore, we concluded that our ACE keyword list improved upon the published one and continued to use the former in the following analyses.

Intriguingly, the ACE alignment index tended to be larger for individuals sampled using the non–ACE-1 keyword list than for those sampled using the non–ACE-2 keyword list (mean 0.44, SD 0.09 for non–ACE-1 and mean 0.36, SD 0.13 for non–ACE-2; non–ACE-1 vs non–ACE-2: *P*<.001). In fact, 16.6% (413/2482) of the individuals sampled using the non–ACE-1 keyword list were ACE individuals (ie, ACE alignment index ≥0.5), whereas a substantially smaller fraction of the individuals sampled using the non–ACE-2 keyword list (34/515, 6.6%) were ACE individuals. This result indicates that individuals who tweet about their own parents tend to talk about ACEs more than those who do not. Mainly for this reason, we needed to sample many Twitter users using the non–ACE-1 keyword list to be able to sample non-ACE individuals, which are defined as those whose maximum ACE mention score in their tweets is <0.3. We identified 4.79% (119/2482) and 20.6% (106/515) of non-ACE individuals using the non–ACE-1 and non–ACE-2 keyword lists, respectively.

### Sentiment and Content of Tweets

**Figure 4:**
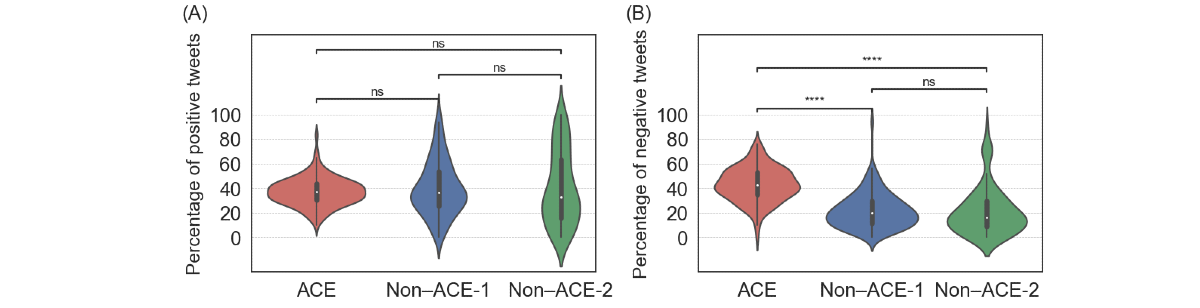
Distribution of the percentage of positive and negative tweets for adverse childhood experience (ACE) and non-ACE individuals. (A) Percentage of positive tweets. (B) Percentage of negative tweets. We used the violin plots implemented in Seaborn, a Python library (Python Software Foundation), to visualize the distributions. The open circle in each violin plot represents the median. The thick vertical lines represent the IQR. The thin vertical lines represent the range. ****P<.001 based on the 2-sided Mann-Whitney-Wilcoxon test with Bonferroni correction; ns: not statistically significant.

We analyzed sentiments of the tweets posted by the ACE, non–ACE-1, and non–ACE-2 root users. We show the distributions of the percentages of positive tweets and of negative tweets for each of the 3 groups of root users in Figure 4. We found that ACE individuals posted negative tweets more frequently than non-ACE individuals and that there was no statistically significant difference in the fraction of positive tweets they posted (*P*=.99).

We also ran a content analysis on the collected tweets using the latent Dirichlet allocation, which is a standard topic modeling technique for carrying out content analysis. We found that each topic identified for the set of tweets posted by the ACE root users contained words related to traumatic experiences and that no topic identified for the set of tweets posted by the non–ACE-1 and non–ACE-2 root users contained such words (see the *Content Analysis* section in Multimedia Appendix 1 for results). This result further justifies our CNN-based classifier.

### Analysis of Egocentric Networks of ACE and Non-ACE Individuals

#### Number of Followers and Followees

**Figure 5:**
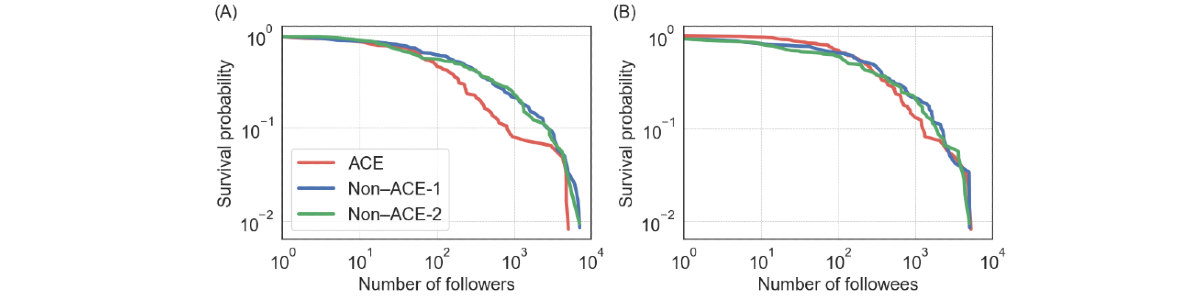
Survival probability of the distribution of the number of follow edges. (A) Number of followers. (B) Number of followees. ACE: adverse childhood experience.

For the egocentric networks of the root users, we first investigated the number of followers and followees for each type of root user. We show the survival probability of the number of followers, *k*^in^ (ie, the fraction of the root users whose number of followers is at least *k*^in^), in Figure 5A. Each distribution has a heavy tail, which is typical for distributions of the number of followers or followees on Twitter [53]. In other words, a small fraction of individuals has disproportionately many followers or followees compared with the majority. The number of followers was only statistically significant different between the ACE and non–ACE-1 groups (ACE vs non–ACE-1: *P*=.03; ACE vs non–ACE-2: *P*=.20; non–ACE-1 vs non–ACE-2: *P*=.93), with *k*^in^ being smaller for the ACE than for the non–ACE-1 group. However, the statistically nonsignificant result in the comparison of the ACE and non–ACE-2 groups was presumably due to the large SDs. In fact, their average *k*^in^ values were substantially different from each other (mean 433.8, SD 1058.9, and *k*^in^ range 0-5046 for ACE; mean 795.2, SD 1413.9, and *k*^in^ range 0-7089 for non–ACE-1; mean 764.3, SD 1335.6, and *k*^in^ range 0-6977 for non–ACE-2). Overall, ACE individuals tended to have fewer followers than non-ACE individuals, which is also notable in Figure 5A. This result may be because ACE individuals’ tweets attract less people than the tweets by non-ACE individuals on average. In contrast, the distribution of the number of followees, *k*^out^ was similar among the 3 groups (Figure 5B; mean 551.6, SD 993.9, and *k*^out^ range 1-5213 for ACE; mean 687.6, SD 1080.3, and *k*^out^ range 0-5035 for non–ACE-1; mean 605, SD 1039.7, and *k*^out^ range 0-5000 for non–ACE-2; ACE vs non–ACE-1: *P*=.93; ACE vs non–ACE-2: *P*=.39; non–ACE-1 vs non–ACE-2: *P*=.69).

#### Reciprocity

Next, to inspect possible differences in the structure of the egocentric network between ACE and non-ACE individuals, we investigated the reciprocity (ie, the fraction of mutual following between 2 individuals), local clustering coefficient (ie, the abundance of triangles), and homophily (ie, the fraction of ACE users in the immediate neighborhood of an ACE or non-ACE individual) for the root users.

**Figure 6:**
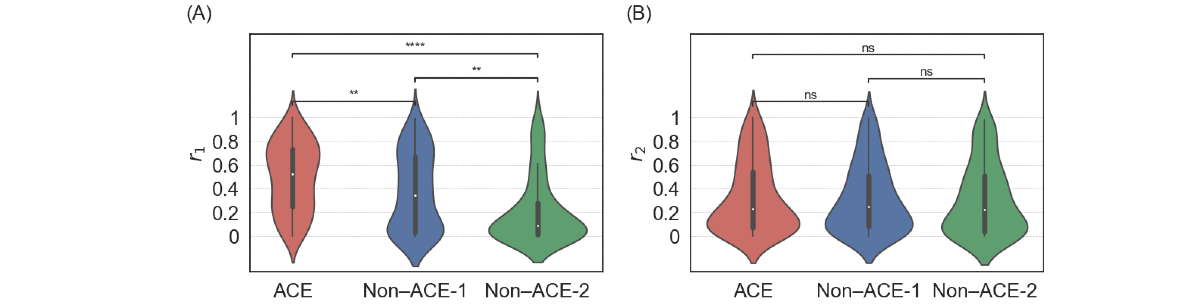
Distribution of the reciprocity for adverse childhood experience (ACE) and non-ACE individuals. (A) *r*1. (B) *r*2. **P<.01 and ****P<.001 based on the 2-sided Mann-Whitney-Wilcoxon test with Bonferroni correction; ns: not statistically significant.

We show the distribution of 2 types of reciprocity, *r*_1_ and *r*_2_, separately for the 3 groups of root users as violin plots in Figure 6. The *r*_1_ value was statistically significantly different among the 3 groups. Specifically, *r*_1_ for the ACE group was larger than that for the non–ACE-1 group (*P*<.01), which was larger than that for the non–ACE-2 group (*P*<.01). We did not find statistically significant differences among the 3 groups in terms of *r*_2_ (*P*=.99). These results indicate that ACE individuals tend to follow back their followers, no matter which individual first started to follow the other, and that the individuals followed by a root ACE user do not particularly tend to follow back the root ACE user. Therefore, ACE individuals may tend to proactively connect with other individuals to yield reciprocal follow edges.

#### Local Clustering Coefficients

**Figure 7:**
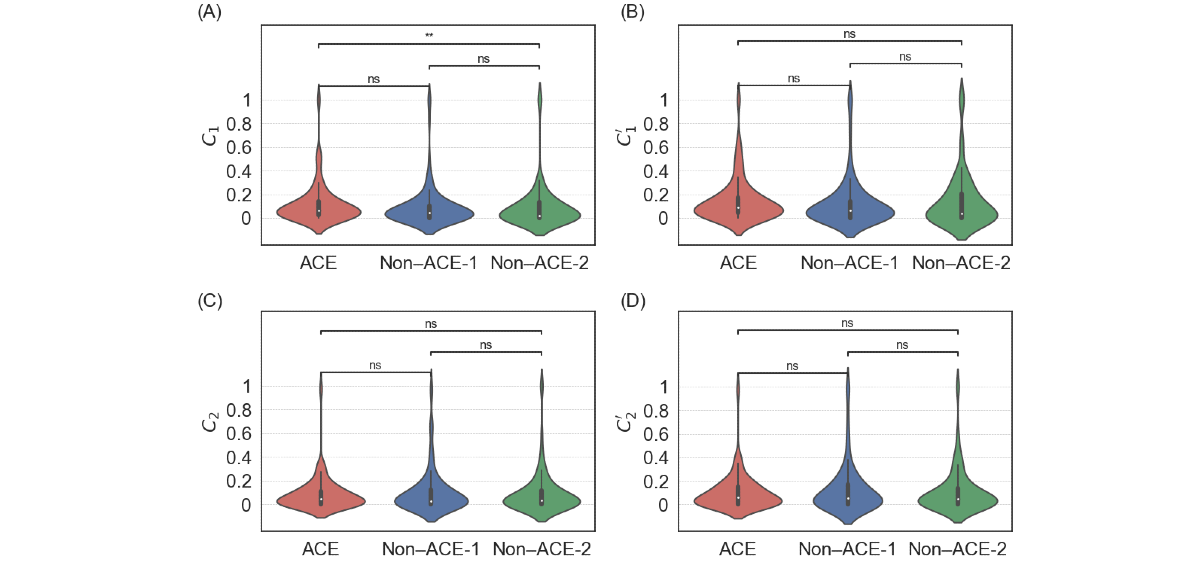
Distribution of the local clustering coefficient for adverse childhood experience (ACE) and non-ACE individuals. (A) *C*_1_. (B) *C*_1_^*'*^. (C) *C*_2_. (D) *C*_2_^*'*^. **P<.01 based on the 2-sided Mann-Whitney-Wilcoxon test with Bonferroni correction; ns: not statistically significant.

We show the distributions of the local clustering coefficients for the ACE and non-ACE groups in Figure 7. The computation of the local clustering coefficient requires sampling of reciprocal neighbors of the root users *u*. When we collected *u*’s reciprocal neighbors by examining whether *u* followed back their followers, we only found statistically significant results between the ACE and non–ACE-2 groups for the stronger definition of the local clustering coefficient, as shown in Figure 7A (*P*=.002 for *C_1_*). There was no statistically significant difference between the ACE and non–ACE-2 groups for the weaker definition of local clustering coefficient, as shown in Figure 7B (*P*=.06 for *C_1_^’^*). In particular, there was no difference between the ACE and non–ACE-1 groups (*P*=.07 for *C*_1_; *P*=.12 for *C_1_^’^*). When we collected *u*’s reciprocal neighbors by examining whether *u*’s followers followed *u* back, we did not find statistically significant results between any pair of groups either for the stronger or weaker definitions of the local clustering coefficient (ie, *C_2_* and *C_2_^’^*; Figures 7C and 7D, respectively; *P*=.99 for all cases). Overall, we concluded that the abundance of triangles around the root users, as quantified by the local clustering coefficients, was not different among the 3 groups in most cases.

#### Homophily

**Figure 8:**
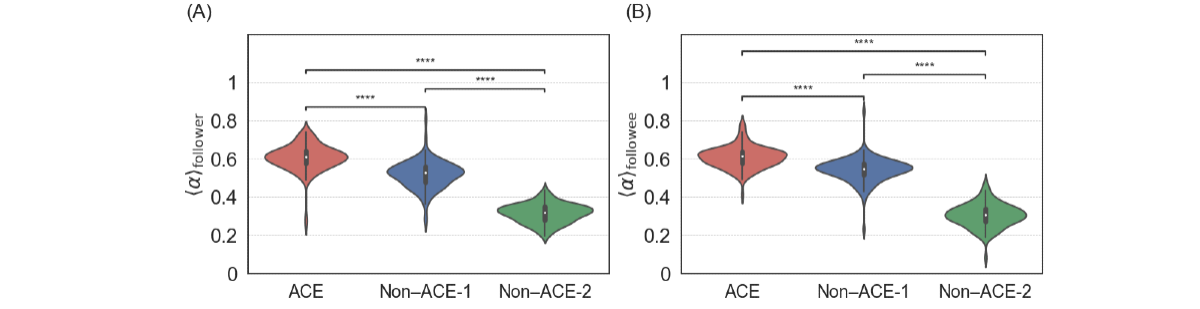
Distribution of the average adverse childhood experience (ACE) alignment index over the followers and followees of the ACE and non-ACE root users. (A) Average ACE alignment index over the followers, 〈α〉_follower_. (B) Average ACE alignment index over the followees, 〈α〉_followee_. ****P<.001 based on the 2-sided Mann-Whitney-Wilcoxon test with Bonferroni correction.

The homophily in terms of ACEs would be a tendency for ACE individuals to be preferentially connected with other ACE individuals in the network. To investigate the possibility of such homophily, we measured the average ACE alignment index of the followers, denoted using 〈α〉_follower_, of each ACE and non-ACE root user. We show the distribution of 〈α〉_follower_ for the different groups of root users in Figure 8A. The ACE alignment index for the followers of ACE individuals was statistically significantly larger than that for the followers of non-ACE individuals (*P*<.001), supporting the homophily hypothesis. We also found that non–ACE-1 individuals had a statistically significantly larger 〈α〉_follower_ than non–ACE-2 individuals (*P*<.001). We then measured the average ACE alignment index of the followees, denoted using 〈α〉_followee_, of each root user. The distributions of 〈α〉_followee_ for the ACE, non–ACE-1, and non–ACE-2 groups of root users, shown in Figure 8B, were similar to those of 〈α〉_follower_ shown in Figure 8A, including the statistical results (all *P*<.001).

Figure 6 supports that ACE individuals tend to follow back their followers. Figure 8 supports that ACE individuals, as compared with non-ACE individuals, are more likely to be directly connected with other ACE individuals. The combination of these 2 results suggests that ACE individuals are more likely to follow back their ACE followers than their non-ACE followers. Therefore, we conducted a subanalysis to compare the average ACE alignment index of the reciprocal neighbors of root users *u* and of the nonreciprocal followers of *u*.

**Figure 9:**
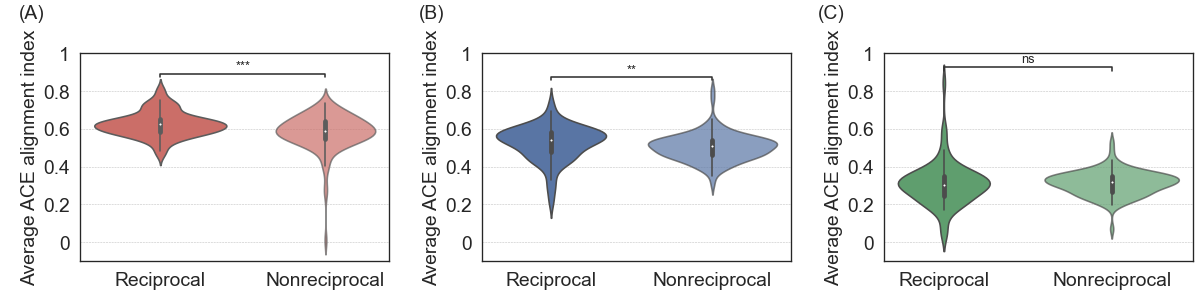
Distribution of the average adverse childhood experience (ACE) alignment index over the reciprocal neighbors and the nonreciprocal followers. (A) ACE root users. (B) Non–ACE-1 root users. (C) Non–ACE-2 root users. **P<.01 and ***P<.001 based on the 2-sided Mann-Whitney-Wilcoxon test with Bonferroni correction; ns: not statistically significant.

We show the distribution of the average ACE alignment index of the reciprocal neighbors and the nonreciprocal followers in Figures 9A, 9B, and 9C for ACE, non–ACE-1, and non–ACE-2 root users, respectively. The results statistically support that the ACE and non–ACE-1 root users, in particular the ACE root users, tended to follow back ACE individuals more than non-ACE individuals, whereas the difference in the average ACE alignment index between the reciprocal neighbors and nonreciprocal followers was small. The difference between the reciprocal neighbors and nonreciprocal followers was not statistically significant for the non–ACE-2 root users (*P*=.28). Note that this result that non–ACE-1 root users behave more similarly to ACE root users than non–ACE-2 root users do is consistent with our results for reciprocity (Figure 6), local clustering coefficient (Figures 7A and 7B), and homophily (Figure 8).

## Discussion

### Principal Findings

We trained a CNN model with Reddit post title data to classify tweets on Twitter into those associated with ACEs and those not. Using the trained CNN as a main tool, we sampled Twitter users, determined their strength of association with ACEs in terms of our ACE alignment index, and investigated the structure of egocentric follow networks of individuals reporting ACEs and those not reporting ACEs. We found that individuals reporting ACEs, compared with non–ACE-reporting individuals, have fewer followers, similar numbers of followees (ie, other individuals that an individual follows), a higher propensity to follow back, similar abundance of triangles around the individual (ie, similar local clustering coefficient values), more ACE individuals as their followers and followees, and a higher tendency to follow back other individuals reporting ACEs rather than other individuals not reporting ACEs. Social networks of individuals reporting ACEs have been largely unknown except for the number and quality of their immediate contacts. This study substantially expands our understanding of their social networks by combining machine learning techniques, modern network analysis, and web-based social media data.

Studies suggest that connectedness through social networks can support individuals who have experienced ACEs [16,17] or various mental health challenges [54] and confer a protective effect by disrupting the toxic stress pathways that connect adversity and trauma in childhood with poor health outcomes across the life span [19,55]. The connectedness and social support that may emerge from social networks can also provide a positive adaptation and coping strategy, thereby reducing the need for maladaptive health risk behaviors to cope with the impact of ACEs. These studies and ours share the main goal of revealing characteristics about the networks around individuals reporting ACEs. In 1 study [16], the authors quantified the social networks using the number of other individuals and social groups to which the participants belonged. In contrast, in addition to the number of directly connected other individuals, we examined the association between ACEs and further structural properties of the individuals’ social networks, that is, the reciprocity of edges, local abundance of triangles, and homophily (ie, the relative frequency with which ACE individuals follow other ACE individuals). In particular, we found that individuals reporting ACEs tend to make reciprocal follows and that they do so more frequently with other ACE individuals. This result may indicate that ACE individuals are actively seeking social support by connecting with other ACE individuals to share their ACEs and current lives. Existing strategies to prevent and respond to ACEs in trauma-informed ways take place in clinical, family, school, community, and some institutional settings [2]. Our results suggest the possibility of an additional venue—social networks and social media platforms—for prevention and mitigation strategies. This possibility has the added benefit of being more universally available, although attention to rural communities is needed, and accessible, particularly for those reluctant to seek support in more traditional and visible ways. Investigating the efficacy of this method warrants future work.

We found that Twitter users who mention their parents using the word *my* (eg, *my mother*) tend to be more likely associated with ACEs than those who do not. This result is supported by the different distributions of the ACE alignment index, reciprocity, and homophily between the non–ACE-1 and non–ACE-2 groups. We cannot rule out the possibility that this result was solely because users in the non–ACE-1 group tended to talk about their own parents, which may have led to more misclassification of their tweets. However, this information may be useful for detecting and documenting ACEs in people’s web-based posts and questionnaire correspondences. Furthermore, systematically investigating whether people tend to use those words (eg, *my father*) with negative meanings and sentiments when they use them, in particular on social media, is an intriguing research question.

Previous research has shown that individuals with major depression [25,26], suicide ideation [25,28], or anxiety disorder [29] tend to have smaller local clustering coefficients (ie, less triangles around the individuals) than healthy controls. As triangles in networks are positively associated with social support [28,50,51], these results may indicate that the affected individuals are lacking in social support. In contrast, a different study using Twitter data showed that individuals with depression tended to have a higher local clustering coefficient than controls, suggesting that individuals with depression may prefer to build a closed network in which they want to share their experiences and obtain social support [22]. In contrast to these studies, we did not find differences in the local clustering coefficients between the ACE-reporting and non–ACE-reporting individuals. The reason for this result is unclear. However, it does not contradict our interpretation of the main results of this study that ACE individuals’ high reciprocity and homophily in following behavior may reflect the social support that they seek (ie, connection with other ACE individuals). Investigating the nature of triangles, such as who is in the triangle, requires more data, such as exhaustive sampling of the followers and followees of the root users. However, these tasks are computationally difficult because of the rate limit imposed by Twitter. Future work using different social media and other types of data containing information about triangles may be able to better understand this topic.

People with traumatic experiences may be less likely to speak about them in public because of stigma, that is, to avoid the label of a mental health problem; with the label, the public may be reluctant to accept them [56,57]. Our result that ACE-reporting individuals tended to have less followers may be a consequence of such a reluctance of the public. By definition, individuals with ACEs who do not tweet about their ACEs should have low ACE alignment index values. In fact, these individuals may preferentially follow other ACE individuals. If this is the case, the difference between the average ACE alignment index for the followers of ACE-reporting and non–ACE-reporting individuals, shown in Figure 8A, may be an underestimation. Combining classification methods for ACEs based on social media posts, such as the one used in this work, and those based on other digital data, such as passive sensing [58,59], may help us improve the accuracy of the classification and explore the possibly different behavior of ACE-reporting individuals who openly talk about their experiences and those who do not.

We used Reddit and Twitter data to sample and assess individuals reporting ACEs. There are at least 2 strengths of this approach compared with conventional questionnaire-based methods. First, the data used are observational. As Reddit and Twitter users’ posting behavior is unrestricted by experimenters, the obtained data are expected to be less subject to recall and other biases, which data collection based on retrospective reporting is generally subject to [60-62]. A second strength is scalability. Although the free Twitter API is rate-limited, one can still collect information about tweets and follow relationships of many individuals without much difficulty.

### Limitations

In contrast to their strengths, a potential main drawback of social media data is credibility. For example, a previous study pointed out that credibility and quality of tweet data about ACEs are not guaranteed [20]. Another clear limitation of Twitter and some other social media data is that data are poorly annotated. For example, our results may be confounded by nuisance parameters such as the individual’s age, sex, education, geographical location, economic status, and race. Many people do not reveal such information on Twitter, and there is no personal information on users’ public profiles on Reddit. Therefore, part of the statistically significant differences among the 3 groups revealed in this study may be due to unmeasurable factors such as the different demographic characteristics of the 3 groups. This limitation made it difficult to define the control groups in this study, and thus, we used 2 non-ACE groups (ie, non–ACE-1 and non–ACE-2). The lack of annotation also made it difficult to estimate the severity of ACEs, such as the standard ACE score [63] for Twitter users. There are clear trade-offs between the advantages of social media data and of questionnaire-generated data. Validating these results with clinical populations, such as by asking them about specific uses of Twitter or other social media, may benefit both social media– and questionnaire-based studies of ACEs.

There are also other limitations of this study. First, we used Twitter’s follow network as a proxy to social interaction between individuals. However, follow behavior, including the case of reciprocal following, does not necessarily represent a friendship or reasonable social relationship [51,64]. Future studies should look into other social media networks or combine social media data and questionnaire data to better estimate social networks, including the meaning of the edge (ie, link) of the network.

Second, there may be transferability issues between the Reddit and Twitter data. In fact, the accuracy of the CNN on the Twitter data was high as long as we sampled some ACE and non-ACE Twitter users and manually inspected their tweets. However, users on Reddit, which our CNN was trained on, may be statistically different from Twitter users in terms of demography. It is also likely that people using both Reddit and Twitter often publish Reddit posts and tweets with different intentions and in different situations. Although transfer learning is a common technique in data science, it is desirable to enhance the homogeneity of the 2 populations through better sampling and user profiling.

Third, there is no established approach to ACE-related search terms on Twitter data. We defined our search terms via a combination of literature [20] and knowledge of experts in our team. However, public discourse and social media users may not use the same language to refer to childhood trauma and adversity as experts. Therefore, we may have missed individuals reporting ACEs on Reddit and Twitter using their own terms. Thorough text analyses of posts in web-based ACE communities are expected to help better definitions of search terms for similar studies in the future.

Fourth, one may be able to improve the accuracy of our CNN using a different type of classifier or a larger amount of training data, including those from different subreddits and other social media platforms relevant to ACEs. In fact, our CNN is not specialized to NLP, and we showed that the BERT, a classifier specialized to NLP, did not improve the classification performance (see the *Performance of the CNN Classifier* section). Despite these limitations, we believe that this study clarifies new features of social networks with people with ACEs and suggests opportunities for future research on ACEs involving social media data, network analysis, and machine learning techniques.

## Appendix

Multimedia Appendix 1 Analysis of Twitter data using the Bidirectional Encoder Representations from Transformers (BERT) classifier and content analysis.

## Abbreviations

ACE: adverse childhood experience
API: application programming interface
AUC: area under the receiver operating characteristic curve
BERT: Bidirectional Encoder Representations from Transformers
CNN: convolutional neural network
FN: false negative
FP: false positive
GloVe: Global Vectors for Word Representation
NLP: natural language processing
RegEx: regular expression
TN: true negative
TP: true positive
TWINT: Twitter Intelligence Tool
VADER: Valence Aware Dictionary and Sentiment Reasoner
