# Supplementary material for: Web-Based Social Networks of Individuals With Adverse Childhood Experiences: Quantitative Study
Source: J Med Internet Res. 2023 May 30;25:e45171. doi: 10.2196/45171 (PMC10265411; doi:10.2196/45171)
Supplement: Multimedia Appendix 1 [file jmir_v25i1e45171_app1.docx]

**Supplementary Information for:**

**Online social networks of individuals with adverse childhood experiences**

Yiding Cao MA, Suraj Rajendran^+^ BS, Prathic Sundararajan^+^ BS,

Royal Law PhD MPH, Sarah Bacon PhD, Steven A. Sumner MD MSc, Naoki Masuda^∗^ PhD

+ Equal contribution

* Corresponding author: naokimas@buffalo.edu

**S1 Analysis of Twitter data using the BERT classifier**

We calculated the ACE alignment index for all the users sampled with the ACE, non-ACE-1, and non-ACE-2 keyword lists using the BERT classifier. We identified 117 ACE root users out of the 126 users sampled with the ACE keyword list, 123 non-ACE-1 root users out of the 2482 users sampled with the non-ACE-1 keyword list, and 109 non-ACE-2 root users out of the 515 users sampled with the non-ACE-2 keyword list. Most of the ACE root users identified using BERT are identical with those identified using CNN, and the same holds true for the non-ACE users. See Table S1 for the number of the ACE and non-ACE users identified with both BERT and CNN, just one of them, or neither. The Jaccard index measuring the overlap of the root users identified by CNN and BERT is equal to 0.920, 0.911, and 0.904 for the ACE, non-ACE-1, and non-ACE-2 groups, respectively; the Jaccard index between sets $A$ and $B$ is given by $J\left( A,B \right)= \left| A\cap B \right|/\left| A\cup B \right|$. Because more than 90% of ACE and non-ACE users overlap between CNN and BERT, the results of the network analysis obtained with BERT are expected to be similar to those obtained with CNN.

Table S1: Number of ACE and non-ACE root users identified with CNN or BERT.

|  | CNN *and* BERT | CNN *and not* BERT | BERT *and not* CNN |
| --- | --- | --- | --- |
| ACE | 115 | 8 | 2 |
| Non-ACE-1 | 113 | 6 | 5 |
| Non-ACE-2 | 103 | 3 | 8 |

**S2 Content analysis**

A main task of content analysis is to categorize a set of text into a limited number of themes or topics based on the words in the text [1]. Traditional content analyses, which involve manual scanning of the documents to search the words, are not scalable [2]. Our study uses a large size of text, i.e., 23,542, 29,755, and 37,433 tweets posted by ACE, non-ACE-1, and non-ACE-2 root users, respectively. Therefore, we applied the latent Dirichlet allocation (LDA), which is a topic modeling technique widely used for content analysis for extracting topics from large volumes of documents [2, 3, 4].

The first step of topic modeling is to determine the optimal number of topics of the given set of text. A reliable method to do this is to compute the topic coherence for different numbers of topics and select the number of topics using the elbow method [5]. For example, Fig. S1 shows that the optimal number of topics, corresponding to the highest coherence score, is 5, 9, and 7 for the ACE, non-ACE-1, and non-ACE-2 groups, respectively. We show in Tables S2, S3, and S4 the topics extracted from tweets posted by the ACE, non-ACE-1, and non-ACE-2 root users, respectively. Table S2 indicates that every topic of tweets posted by the ACE root users includes words related to traumatic experiences, such as abuse, narcissist, assault, and trauma. In contrast, the topics of the tweets posted by non-ACE root users do not contain words related to traumatic experiences (see Tables S3 and S4).


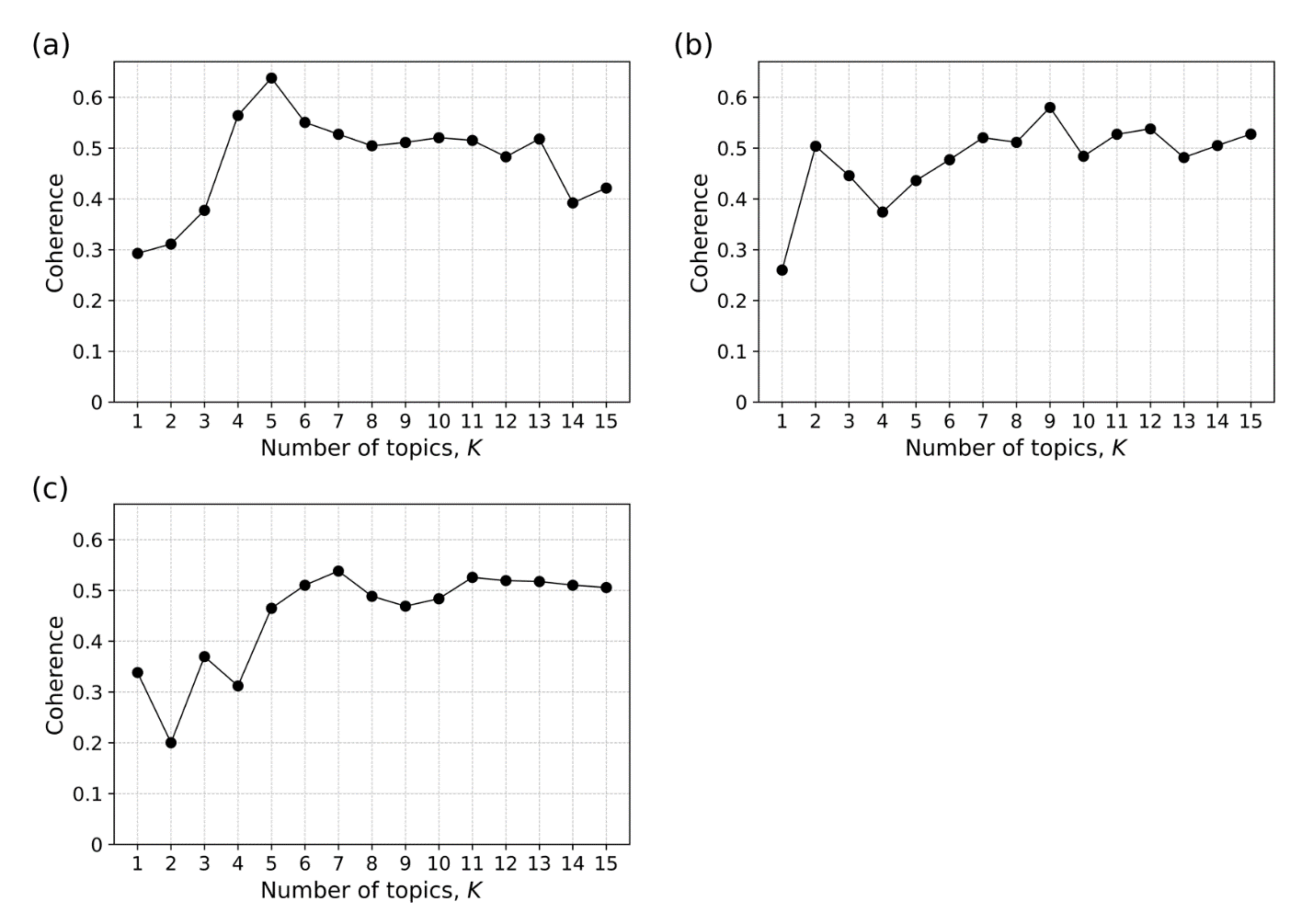


Figure S1. Topic coherence score for the tweets calculated for the LDA with the number of topics *K* = {1, ..., 15}. (a) ACE root users. (b) Non-ACE-1 root users. (c) Non-ACE-2 root users.

Table S2: Topics of the tweets posted by the ACE root users and the associated frequent words. “No.” stands for number. The percentages in the parentheses show the fraction of the tweets belonging to the specified topic among all the tweets posted by the ACE root users. Similar conventions apply to Tables S3 and S4.

| Topic | No. of tweets | Top 10 words |
| --- | --- | --- |
| 1 | 5,211 (22%) | assault, hell, false, abuse, sex, women, world, war, know, thanks |
| 2 | 3,697 (16%) | abuser, lies, case, going, said, victim, proof, contact, amberheard, johnnydepp |
| 3 | 4,445 (19%) | life, work, shit, trauma, said, fuck, love, want, hate, time |
| 4 | 5,316 (23%) | think, abuse, people, parents, say, child, years, need, way, feel |
| 5 | 4,873 (20%) | mom, family, origin, narcissist, abuse, shit, contact, better, thought, personality |

Table S3: Topics of the tweets posted by the non-ACE-1 root users and the associated frequent words.

| Topic | No. of tweets | Top 10 words |
| --- | --- | --- |
| 1 | 3337 (11%) | kit, mother, black, suits, thinking, jacket, new, listing, blue, birthday |
| 2 | 5268 (18%) | date, money, photo, best, posted, online, women, need, know, share |
| 3 | 3671 (12%) | chunky, cheese, figure, ear, dinner, milk, contact, sick, tonight, earn |
| 4 | 2911 (10%) | air, like, winter, halloween, blue, car, diy, holiday, dad, christmas |
| 5 | 1751 (6%) | notifications, boy, bell, page, playing, follow, tutorial, son, make, cat |
| 6 | 4985 (17%) | sister, grandson, wife, salary, finish, round, son, career, white, diamond |
| 7 | 2253 (8%) | price, published, update, change, carry, bank, lower, bought, monitoring, day |
| 8 | 2199 (7%) | stay, county, varsity, game, family, live, high, team, stream, home |
| 9 | 3374 (11%) | google, radio, god, lifeline, dog, productions, contact, lord, western, tags |

Table S4: Topics of the tweets posted by the non-ACE-2 root users and the associated frequent words.

| Topic | No. of tweets | Top 10 words |
| --- | --- | --- |
| 1 | 3163 (8%) | baseball, varsity, team, today, softball, playoff, live, neutral, churchill, oh |
| 2 | 4328 (12%) | companies, business, twitch, new, bipolar, check, year, mobile, scapegoat, ebay |
| 3 | 3578 (10%) | area, driver, speed, way, playing, travel, news, com, old, road |
| 4 | 8450 (22%) | listen, best, enjoy, know, good, hear, comedy, random, link, follow |
| 5 | 8471 (23%) | amazon, qualifying, gaming, app, free, purchase, download, pulse, line, edge |
| 6 | 5617 (15%) | music, like, great, device, help, feel, feeling, twitter, online, mood |
| 7 | 3817 (10%) | contact, follow, police, new, make, video, like, deals, shelf, money |

**References**

[1] P. A. Cavazos-Rehg, M. J. Krauss, S. Sowles, S. Connolly, C. Rosas, M. Bharadwaj, & L. J. Bierut. A content analysis of depression-related tweets. *Comput. Hum. Behav.*, *54*: 351–357, 2016.

[2] M. Altaweel, C. Bone, & J. Abrams. Documents as data: A content analysis and topic modeling approach for analyzing responses to ecological disturbances. *Ecol. Inform.*, *51*: 82–95, 2019.

[3] L. Hagen. Content analysis of e-petitions with topic modeling: How to train and evaluate LDA models?. *Inf Process Manag.*, *54*(6): 1292–1307, 2018.

[4] W. X. Zhao, J. Wang, Y. He, J. Y. Nie, J. R. Wen, & X. Li. Incorporating social role theory into topic models for social media content analysis. *IEEE Trans Knowl Data Eng.*, *27*(4): 1032–1044, 2014.

[5] S. Syed, & M. Spruit. Full-text or abstract? examining topic coherence scores using latent dirichlet allocation. *In Proc. Int. Conf. Data Sci. Adv. Anal*., pages 165–174, 2017.
